# Post-surgical Hepatic Herniation: An Exceptionally Rare Occurrence

**DOI:** 10.7759/cureus.84364

**Published:** 2025-05-18

**Authors:** Muhammad Ahmad Mukhtar, Aeimen Khalid, Amna Mukhtar, Rubina Mukhtar

**Affiliations:** 1 General Medicine, York District Hospital, York, GBR; 2 Radiology, Nishtar Medical University, Multan, PAK; 3 General Medicine, Peterborough City Hospital, Peterborough, GBR; 4 General Medicine, Nishtar Hospital, Multan, PAK; 5 Internal Medicine, University of Debrecen, Debrecen, HUN; 6 Radiology, Minar Cancer Hospital, Nishtar Medical University, Multan, PAK

**Keywords:** abdominal hernia, breast carcinoma, conservative management, hepatic herniation, inscisional hernia, post surgical hernia

## Abstract

Post-surgical hepatic herniation through an abdominal incision is an uncommon occurrence, particularly without predisposing factors such as trauma, multiple prior surgeries, or increased intra-abdominal pressure. Complications, such as hepatic Encephalopathy and Budd-Chiari syndrome, are associated with this entity; when they occur, they can cause significant distress. This report describes a 59-year-old female with a history of breast cancer and prior cholecystectomy, in whom a routine CT scan incidentally revealed liver herniation through a surgical scar. She was asymptomatic with normal liver function, and conservative management with regular follow-ups was chosen. CT imaging remains the gold standard for diagnosis. Surgical intervention is reserved for symptomatic or complicated cases. Given the rarity of hepatic herniation, further studies are needed to assess long-term outcomes and establish standardized treatment guidelines. This case underscores the importance of routine imaging in oncology follow-ups.

## Introduction

An abdominal or pelvic hernia occurs when an organ or tissue protrudes through a weakness or defect in the abdominal wall. When this happens at the site of a previous surgical incision, it is classified as an incisional hernia. Abdominal types of hernias are not uncommon, with the bowel being the most frequently herniated organ. Liver herniation through an abdominal wall defect is exceptionally rare. As far as we know, this represents just the seventh documented case of hepatic herniation through the anterior abdominal wall in medical literature [1-3].

The majority of reported liver herniation cases are linked to congenital diaphragmatic hernias or those resulting from chest trauma. It is worth mentioning that liver herniation through a diaphragmatic defect is a separate condition, predominantly seen in pediatric patients, and is not the center of attention here [4]. Reports of liver herniation through the anterior abdominal wall post-surgery are rare, with only five previous cases recorded. Among these, one was managed surgically, while the others were treated conservatively [3,5].

## Case presentation

A 59-year-old female patient underwent a computed tomography (CT) scan as part of a routine follow-up for the evaluation of potential liver metastasis. She had been diagnosed with stage II carcinoma of the breast without evidence of metastasis at the time of initial diagnosis. Her treatment included neoadjuvant chemotherapy followed by lumpectomy. Additionally, she had a history of cholecystectomy performed two years prior.

**Figure 1:**
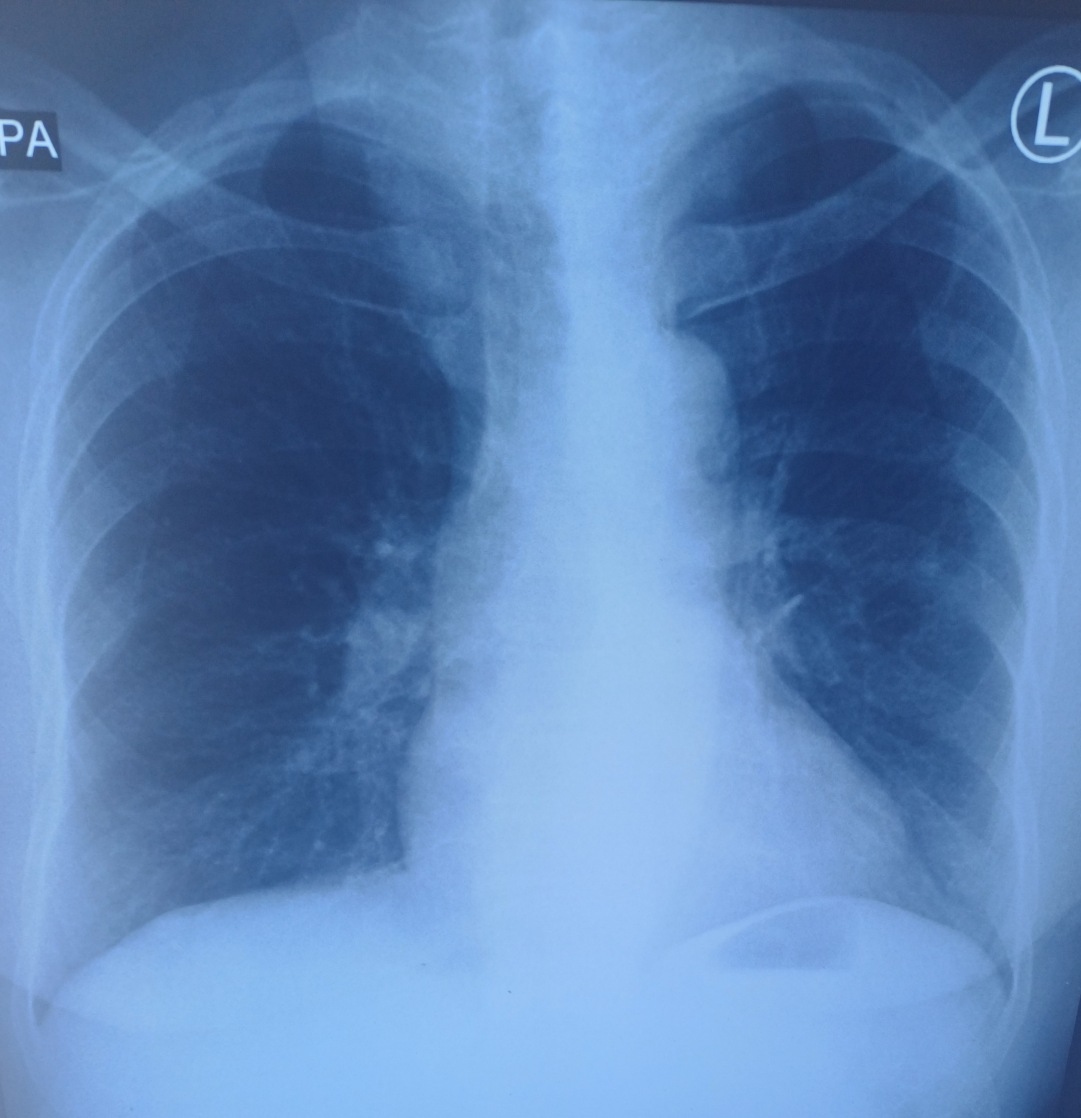
Normal chest radiograph

The patient had no history of predisposing factors for hernia, such as chronic cough, obesity, or previous incisional hernias. She had a normal body habitus with a height of 5 feet 4 inches and a weight of 68 kg. Routine laboratory investigations, including complete blood count (CBC), liver function tests (LFTs) such as serum bilirubin and liver enzymes, and renal function tests (RFTs), were within normal limits (Table 1). A chest x-ray was unremarkable (Figure 1).

**Table 1:** Laboratory results CBC: complete blood count, LFT: liver function test, RFT: renal function test, TLC: total leukocyte count, SGOT: serum glutamic oxaloacetic transaminase, SGPT: serum glutamate pyruvate transaminase

Sr. no	Test		Report	Normal values
1.	CBC	Hb	13	11.5-16.5 g/dL
		TLC	5,600	4,000-11,000/µL
		Platelet count	280	100-400x10^3^/µL
		Neutrophils	64	28-78%
		Lymphocytes	28.4	17-57%
		Monocytes	06	<10%
		Basophils	0.7	<2%
		Eosinophils	0.9	<10%
2.	LFTs	Serum bilirubin	0.6	0.2 -1.0 mg/dL
		Alkaline phosphatase	135	<240 U/L
		SGOT	23	<31 U/L
		SGPT	21	<34 U/L
3.	RFTs	Blood urea	29	10-15mg/dL
		Serum creatinine	1.14	0.7-1.2 mg/dL

**Figure 2:**
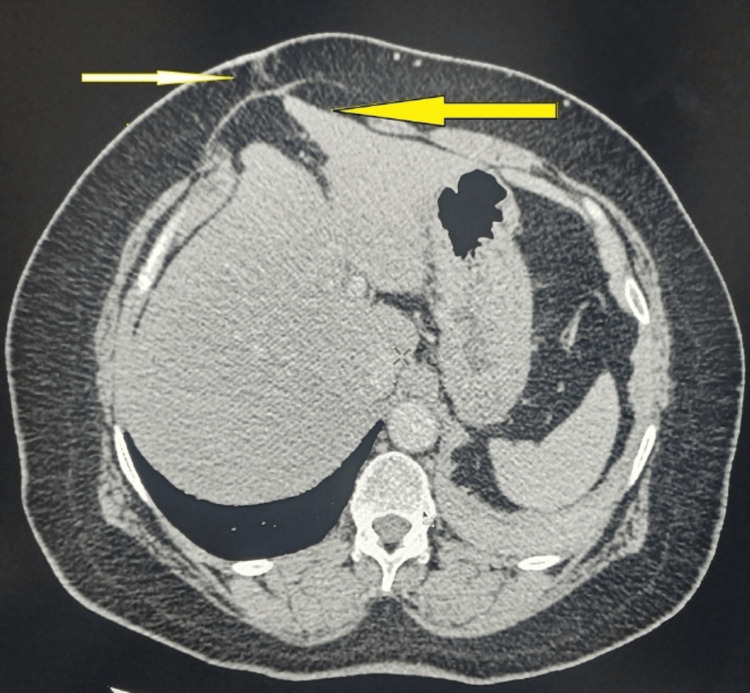
Axial section of CT scan abdomen showing surgical scar with hernial sac in it (white arrow), and herniation of liver at junction of right and left lobe in hernial sac through surgical scar (yellow arrow)

**Figure 3:**
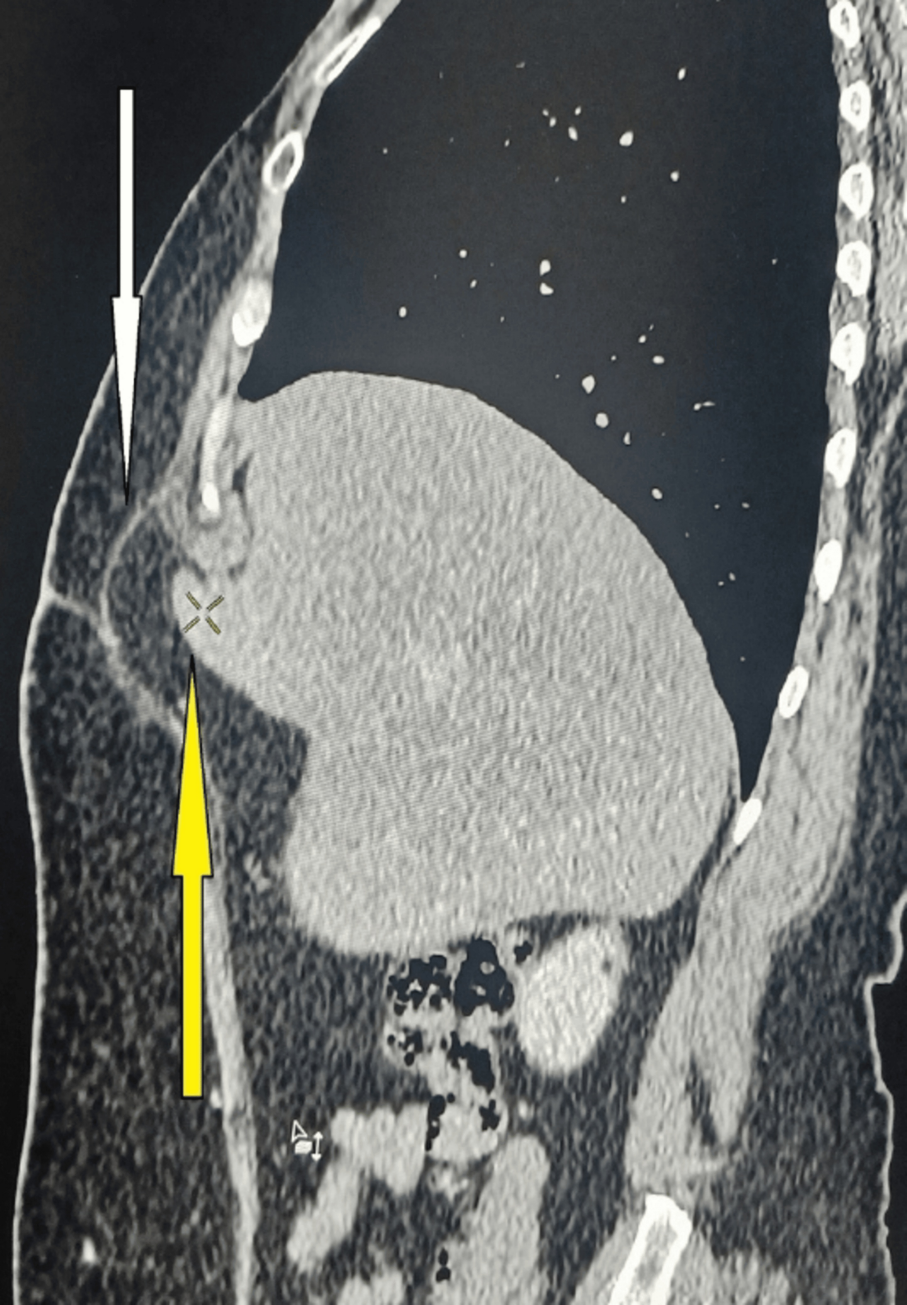
Sagittal section of CT scan abdomen showing hernial sac through surgical scar (white arrow) and herniation of liver into the hernial sac (yellow arrow)

The CT scan incidentally revealed hepatic herniation at the junction of the right and left lobes through a surgical scar in the anterior abdominal wall (Figures 2, 3). The patient remained asymptomatic, with no complaints of pain or discomfort. Given the absence of clinical symptoms and normal liver function, no active surgical intervention was deemed necessary. The patient was placed on conservative management with regular follow-ups at six-month intervals, including clinical evaluation and LFT monitoring, to ensure stability and absence of complications.

In this case, the herniation was incidentally detected during imaging, underscoring the importance of routine surveillance in oncology patients.

## Discussion

Liver herniation through the abdominal wall is a rare occurrence, first documented by Adeonigbagbe et al. in 2000 [6]. Since then, only a few cases have been recorded in medical literature. In comparison, isolated hernias without other physical abnormalities are far more common, with an annual incidence ranging from 2% to 20% [7]. However, liver herniation is an uncommon entity [3,8]. Due to the rarity of hepatic herniation, no specific epidemiologic data exist. However, reported cases have been associated with abdominal incisional hernias, nonalcoholic steatohepatitis, coronary artery bypass grafting (CABG), and direct abdominal trauma [9].

Several risk factors contribute to the development of hepatic herniation through the abdominal wall. These include obesity, advanced age, poor nutritional status, increased intra-abdominal pressure, smoking, weakened abdominal muscles, and post-surgical site infections [9]. Additionally, anatomic variations, such as the congenital absence of the left or right triangular ligaments, have been proposed as potential risk factors. These ligaments anchor the liver to the retroperitoneum, and their absence, in combination with the aforementioned risk factors, may facilitate anterior hepatic herniation [10]. Our patient had a history of prior abdominal surgery and breast carcinoma treatment but lacked other common risk factors.

Clinical presentation and complications

Patients with hepatic herniation may present with a range of symptoms, including abdominal pain, nausea, vomiting, jaundice, dyspnea, confusion, and epigastric swelling [11]. However, many cases, including ours, are diagnosed incidentally through imaging. The clinical implications of hepatic herniation vary depending on the lobe involved.

Left hepatic lobe herniation has been associated with incarceration of the liver within the hernial sac, which can lead to hepatic encephalopathy and liver failure. In one documented case, a patient exhibited elevated hepatic transaminases, flapping tremors, and encephalopathy [12].

Right hepatic lobe herniation has been linked to Budd-Chiari syndrome. One reported case involved a 75-year-old woman who developed secondary Budd-Chiari syndrome decades after a partial nephrectomy. Despite being asymptomatic, imaging confirmed the diagnosis [11,13]. Given the potential morbidity and mortality, clinicians should be vigilant in recognizing this complication.

Diagnosis and management

Hepatic herniation should be suspected in patients presenting with epigastric bulging, but definitive diagnosis requires imaging. CT scanning is the preferred modality for confirming hepatic herniation and evaluating associated complications [3]. In our case, CT imaging performed during a follow-up for liver metastases incidentally revealed the herniation. Ultrasonography in experienced hands might detect it, but due to a lack of accuracy, it is not the modality of choice.

The optimal management of hepatic herniation remains uncertain, as no established treatment guidelines exist. In most cases, conservative management is preferred, particularly for asymptomatic patients. However, surgical intervention is warranted when complications arise. It is important to note that patients with cirrhosis face increased risks of morbidity and mortality following surgical repair of abdominal hernias [3].

Hepatic herniation through the abdominal wall remains exceptionally rare [3]. The majority of reported cases involve congenital diaphragmatic hernias or diaphragmatic rupture following trauma [3]. Acquired liver herniation through the abdominal wall has been documented in only seven adult patients: one without prior surgery, two following sternotomy, and four after abdominal surgery [5,6,14]. To our knowledge, this case represents the seventh report of liver herniation through an abdominal wall defect post-surgery in the English-language literature.

Although conservative treatment is the first-line approach in asymptomatic cases, surgical intervention should be considered for patients with severe symptoms or complications. Given the rarity of this condition, it is difficult to identify definitive predisposing risk factors.

## Conclusions

Hepatic herniation through the abdominal wall is a very uncommon condition with limited documentation in medical literature. Potential complications, including hepatic incarceration and Budd-Chiari syndrome, highlight the importance of early identification through imaging, particularly CT scans. Our case highlights the rare occurrence of hepatic herniation through a surgical scar in an asymptomatic patient. Given the asymptomatic nature of the condition, a conservative approach with regular monitoring was adopted rather than surgical intervention, which is reserved for associated complications. Further studies are needed to evaluate the long-term outcomes of such incidental findings and to develop standardized management protocols for similar cases.
